# Dutasteride nanoemulsion preparation to inhibit 5‐alpha‐hair follicle reductase enzymes in the hair follicle; an ex vivo study

**DOI:** 10.1049/nbt2.12101

**Published:** 2022-10-31

**Authors:** Mehri Memar Bashi Aval, Elham Hoveizi, Reza Mombeiny, Mostafa Kazemi, Saeedeh Saeedi, Shima Tavakol

**Affiliations:** ^1^ Department of Medical Nanotechnology Faculty of Advanced Sciences and Technology Pharmaceutical Sciences Branch Islamic Azad University, (IAUPS) Tehran Iran; ^2^ Department of Biology Faculty of Science Shahid Chamran University of Ahvaz Ahvaz Iran; ^3^ Department of Medical Nanotechnology Faculty of Advanced Technologies in Medicine Iran University of Medical Sciences Tehran Iran; ^4^ Department of Pharmaceutical Chemistry School of Pharmacy Shahid Beheshti University of Medical Sciences Tehran Iran; ^5^ Department of Pharmaceutical Sciences College of Pharmacy University of Nebraska Medical Center Omaha Nebraska USA; ^6^ Cellular and Molecular Research Center Iran University of Medical Sciences Tehran Iran

**Keywords:** Alopecia, dutasteride, nanocarrier, particle size, sustained‐release

## Abstract

Alopecia is a treatable disorder that usually occurs due to high levels of 5‐alpha dihydrotestosterone in hair follicles. To enhance the storage capacity of hair follicles and alleviate the inherent characteristics of dutasteride, 5‐alpha reductase inhibitor, a prolonged‐release nanocarrier was synthesised, and its influence on rat abdomen's skin was investigated. Results showed the lower ratio of S/Co (higher ethanol concentration) increased the hydrodynamic nanocarriers' particle size due to thermodynamic disturbance and Ostwald ripening. In contrast, an increase in surfactant through a decrease in interfacial tension resulted in smaller nanocarriers of 32.4 nm. Moreover, an increase in viscosity had an inverse correlation with the nanoemulsions' particle size. Nanocarriers containing ethanol showed less entrapment efficacy, perhaps due to the rapid dissolution of dutasteride into ethanol during nanoemulsification, while, based on Stokes' equation, the addition of ethanol resulted in smaller particle size and stability of the system. Skin permeation analysis using Franz diffusion cells showed nanocarriers could pass through the skin and release dutasteride for 6 days. In conclusion, the optimum concentration of ingredients is decisive in guaranteeing the ideal particle size, stability, and skin permeation of nanocarriers. The Present dutasteride nanocarrier would promise a prolonged and sustained‐release drug delivery system for Alopecia therapy.

## Graphical abstract

Alopecia is a treatable disorder that, over time, gets to be chronically influencing a noteworthy part of the population. To enhance the storage capacity of hair follicles and alleviate the inherent characteristics of dutasteride, a prolonged‐release nanocarrier was synthesised that could pass through the skin and release dutasteride for more than six days. The synthesised dutasteride nanocarrier would promise a prolonged and sustained‐release drug delivery system for Alopecia therapy.

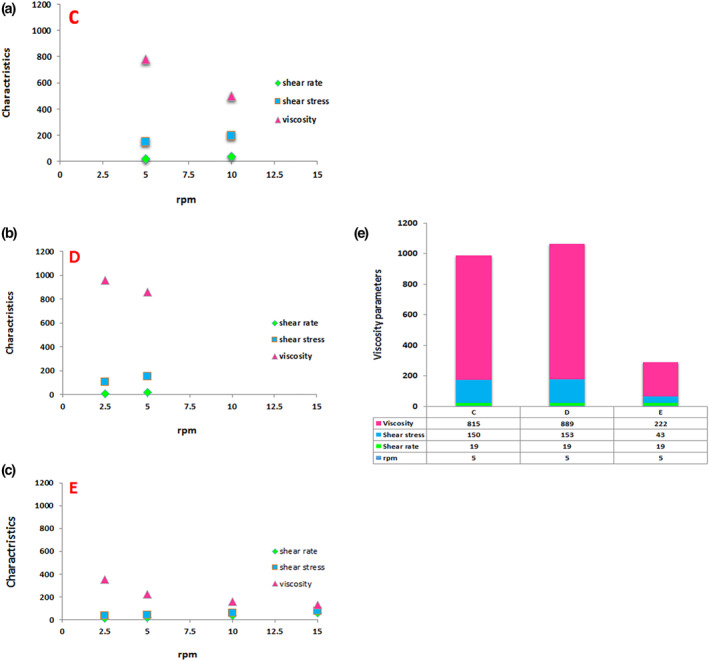

## 1 INTRODUCTION

Because of the evolutionary process, body hair becomes thinner and loses its insulating function in humans. Hair still has this function along with its aesthetic function. Skewering body hair is a remnant of the period when a hairy man pulled his hair together to increase insulation. Research shows that humans lost the hair that covered most of their bodies 3.3 million years ago [1]. The human body has about 5 million hairs, of which 100,000–150,000 are scalp. People with blonde hair have the most significant number of follicles in the head, followed by people with brown hair, red hair, and black hair respectively. Average hair growth is 1 to 0.6 cm per month.

Various factors can affect hair growth and cause permanent or temporary hair loss. Hair loss and thinning have been an issue for men and women throughout history. Finding the cause and treatment of hair loss has become a controversial topic. Scalp hair retains heat and keeps the body warm, protecting the head from sunlight and cold and heat. Hair follicles are divided into two categories depending on androgen and hormones. The follicles on the skull are usually hormone‐dependent. About 3 mm of hair is in the hypodermis. Usually, every 1–4 ends of the hair in the skull and 1–2 fluffy hairs form a follicular unit surrounded by hair straightening muscle [1, 2].

The sebaceous glands target drug treatment in androgenic Alopecia due to the 5‐alpha‐reductase. Hormone 5‐alpha dihydrotestosterone (DHT) is a metabolite of the hormone testosterone. This hormone causes two common diseases in men: benign prostatic hyperplasia (BPH) and male pattern baldness (MPHL) [1, 3]. Both diseases can be successfully treated with a drug that reduces the amount of DHT available to the above organs. These drugs inhibit the activity of an enzyme called 5‐alpha reductase, which converts testosterone to DHT. Dutasteride (Dutasteride), a drug currently under investigation, inhibits the activity of both enzyme forms, type I and type II enzyme 5‐alpha‐reductase. The Food and Drug Administration has recently approved dutasteride for the medical treatment of benign prostatic hyperplasia (BPH) under the brand name Avodart. Inhibition of both types of the enzyme by dutasteride increases this drug's effectiveness in treating male pattern baldness (MPHL). This drug's most effective dose in treating MPHL has been reported to be several times higher than the dose required for treating BPH in Avodart. Type II enzyme in the skin is the predominant form found in hair follicles, while type I is the predominant form of the enzyme found in the sebaceous and sweat glands. The role of type I in the development of MPHL is not yet clear [4]. Hair loss also occurs in women with high levels of 5‐alpha DHT; however, the pattern is different than in men. The occurrence of baldness with high levels of 5‐alpha DHT in hair follicles is due to the effect of DHT on the growth cycle of follicles in the scalp so that increased levels of DHT in hair follicles disrupt the natural hair growth cycle and turn new hair into the hair. Fluffy before total growth (Miniaturisation phenomenon) [5, 6, 7]. Dutasteride is currently available in half‐mg capsules by GlaxoSmithKline company.

Stratum cornea significantly reduces the skin penetration of foreign substances, such as drugs. Therefore, various methods have been considered to modify the drug molecule or its formulation to overcome the stratum corneum barrier [8]. However, the use of microneedles for effective drug penetration has recently been reported [9]. Recently, nanotechnology plays remarkable role in tissue engineering and drug delivery [10, 11, 12, 13]. Studies have shown that nanoparticles can reach deeper parts of the hair follicle in less time, which explains why the infiltration phenomenon can overcome the sebum flow [14, 15] and intracellular infiltration, especially for hydrophobic carriers [1], hydrophilic materials, and high‐molecular‐weight materials are very effective in particle systems [16]. Also, particles of similar size move from the hair cuticle into the hair follicle [17]. According to studies, using a nanocarrier system will help deliver more drugs to the surface below the stratum corneum. Several studies have used a system of solid lipid nanoparticles to deliver materials to hair follicles [18, 19].

The main problem of this system is the large size of the final nanoparticles and less load of a drug into the nanostructure. Another group used liposomes to deliver melanin, which showed follicular delivery [20] but also had a significant size problem compared to the nanoemulsion system. The following is a nanoemulsion system we use in this study. Nanoemulsions provide good thermodynamic stability due to their small particle size. They can also act as a solvent to increase the solubility and thermodynamic activity of the drug and increase the subcutaneous uptake [21]. Some studies show that using ethanol in the structure leads to the dissolution and drying of sebum through the follicular canal and, as a result, will be effective in further drug delivery [22]. Since the storage capacity of hair follicles is up to 10 days [14], we expect the bulk of the drug to be released within 10 days.

## 2 METHODS

### 2.1 Maximum wavelength measurement and standard curve

The maximum wavelength of dutasteride was determined using a UV‐Visible spectrophotometer to draw its standard curve. In brief, 1 mg dutasteride was dissolved within 1 ml of absolute ethanol (Sigma, Germany) to obtain a stock solution of 1 mg/ml stock solution. The absorbance of diluted dutasteride stock solution was measured using the spectrophotometer in the wavelength range of 200–700 nm.

### 2.2 Standard curve

0.0014 g of dutasteride was dissolved within 4.4184 g of ethanol to get a dutasteride's standard curve. The final stock solution (0.25 mg/ml) was stirred at 100 rpm and 25 ± 1.0°C for 30 min. Then, seven serial dilutions were prepared, and their absorbance was read at 242 nm against a blank solution using a UV‐Visible spectrophotometer. The experiment was repeated three times, and a mean ± SD. was reported.

### 2.3 Oil screening

The oil phase must be determined for the nanoemulsion preparation. Therefore, oil screening was performed using three oils: mineral, sesame, and soybean. In brief, 2 mg of dutasteride was added to 1 g of the oils separately. Each solution separately was stirred at 100 rpm and 25 ± 1.0°C for 48 h to achieve equilibrium. After 48 h, samples were centrifuged at 3000 rpm for 15 min, and 10 μl of supernatant was taken and diluted with 200 μl ethanol as the solvent. The absorption was triplicate measured using a UV‐Vis spectrophotometer at 240 nm against a blank solution.

### 2.4 Dutastride nanoemulsion preparation

Dutasteride nanoemulsion oil in water (O/W) was prepared using a low‐energy stirring method. Five hundred micrograms of dutasteride was added to 2000 mg solution, including oil, water, non‐ionic surfactants, and co‐surfactant. Tween 80 (polyoxyethylene Sorbitan monooleate emulsifier (hydrophile)) and Span 80 (sorbitan monooleate emulsifier (lipophile)) were chosen as the surfactants, and ethanol was chosen as the co‐surfactant. In brief, 500 μg dutasteride was added to soybean oil and span 80 as the oil phase. They were stirred for 30 min at room temperature in a dark environment. Then ethanol was added to the oil phase and stirred for an extra 5 min. The water phase included deionised water and Tween 80, in which they were stirred for 30 min. Table 1 shows the percentages of soybean oil, surfactants, and co‐surfactant. The oil phase was added to the water phase at 240 μl/min and stirred at 800 rpm for 30 min. Then, they were characterised for particle size, Potential of hydrogen (pH), thermodynamic stability, etc.

**TABLE 1:** The ingredients of dutasteride nanocarriers. Smic indicates the total percentage of surfactant and co‐surfactant in the formulation, and S/Co indicates the ratio of surfactant/co‐surfactant in the formulation

	Oil (%)	Smic (%)	Co‐surfactant	S/co	Water (%)	Size (nm)	SPAN
A	5	30	Ethanol	01:01	65	5230 ± 13.1	1.20 ± 0.05
B	5	36	Ethanol	02:01	54	4340 ± 15.2	1.19 ± 0.06
C	5	40	Ethanol	03:01	55	32.5 ± 2.12	0.56 ± 0.01
D	10	40	Ethanol	03:01	50	29.5 ± 3.53	0.70 ± 0.02
E	10	30	‐	‐	60	45.4 ± 2.4	0.52 ± 0.01

### 2.5 Nanoparticle characterisation

#### 2.5.1 Particle size and SPAN analysis

A dynamic light scattering instrument determined dutasteride nanocarriers' hydrodynamic particle size and SPAN. In brief, 1 ml of samples was added to the quartz cuvette, and the particle size was measured at the refractive index of 1.523 for 300 s. SPAN was calculated using the formula as follows;

SPAN=D90−D10/D50

The assay was performed in triplicate, and the values provided were the normalised mean ± SD of three independent experiments.

#### 2.5.2 pH analysis

The apparent pH of dutasteride nanoemulsion was measured using a pH metre (Crison, Medidor PH BASIC 20, Spanish) in triplicate at 25°C. The pH of samples was measured in triplicate, and mean ± SD was reported.

#### 2.5.3 Thermodynamic stability evaluation

Thermodynamic stability is one critical characteristic of screening an ideal nanoemulsion. This characteristic of dutasteride nanoemulsion was evaluated in three steps, as follows. The first step was two freeze‐thaw cycles between −20°C (24 h) and room temperature (25°C). The second step was centrifugation at 5000 rpm for 30 min. Then it was followed up by six cycles of heating (40°C) and cooling (4°C) for 48 h. The stable nanoemulsion does not show phase separation or turbidity in each step [19].

#### 2.5.4 Viscosity measurement

The viscosity of dutasteride nanoemulsion was measured using viscometer Brookfield DV‐E with a specific spindle (spindle 42) at room temperature at 2.5, 5, 10, and 15 rpm.

#### 2.5.5 Entrapment efficiency percentage (EE%)

Ultrafiltration is a gold method to evaluate nanocarriers' (Entrapment efficacy [EE%]) [34, 35]. Entrapment efficacy% shows the percentage of entrapped drugs in the nanocarriers. In brief, 500 μl of dutasteride nanoemulsion was added to Amicon ultra‐0.5 ml (molecular weight cutoff 10 kDa). Amicon microtubes were centrifuged for 15 min at 14,000 G. The supernatant was collected, and absorbance was read using a spectrophotometer at 240 nm. The EE% is inversely related to the unentrapped dutasteride in supernatant media. Entrapment efficacy% was calculated using the following formula;

(C−TC/C)×100

C is the total amount of dutasteride, and TC is the free amount of dutasteride, which was detected only in the supernatant media. The assay was performed in triplicate, and the values provided are the mean ± SD of three independent experiments.

### 2.6 Ex vivo study: Permeation analysis

Drug permission analysis on rat skin using a Franz diffusion cell is one of the gold methods to evaluate drug permission through the skin [23, 24, 25]. The method was based on the drug permission method in our published papers [26, 27]. In brief, the receptor cell volume was 6.5 ml, and the effective penetration region was 3 cm^2^. The abdomen skin of the rat was shaved and cut. Then, the fat tissue was removed from the skin pieces, washed with Phosphate buffer saline, and assessed for accuracy. Dutasteride nanoemulsion was poured on the skin in the donor section. A sample of 100 μl was extracted from the section under the skin in the Franz diffusion cell at predetermined time intervals of 20 min, 1 h, 2 h, 4h, 24 h, 48 h, and 6 days. The concentration of released dutasteride was measured using a UV‐Vis spectrophotometer. However, a volume equal to the receptor phase was immediately applied to the Franz diffusion cell to maintain a constant volume.

### 2.7 Statistical data analysis

Graph pad InStat software (V3) was applied to calculate and analyse the dutasteride nanoemulsion's particle size, EE%, and skin permeation. All triplicate experiments were repeated three times. Experiments were performed as mean ± SD. A *p*‐value of less than 0.05 was considered statistically significant.

## 3 RESULTS

### 3.1 Maximum wavelength measurement and standard curve

The maximum wavelength of dutasteride was determined using a UV‐Visible spectrophotometer. Based on the spectra obtained through the spectrophotometer, the maximum absorption wavelength of dutasteride was determined to be 240 nm.

**FIGURE 1:**
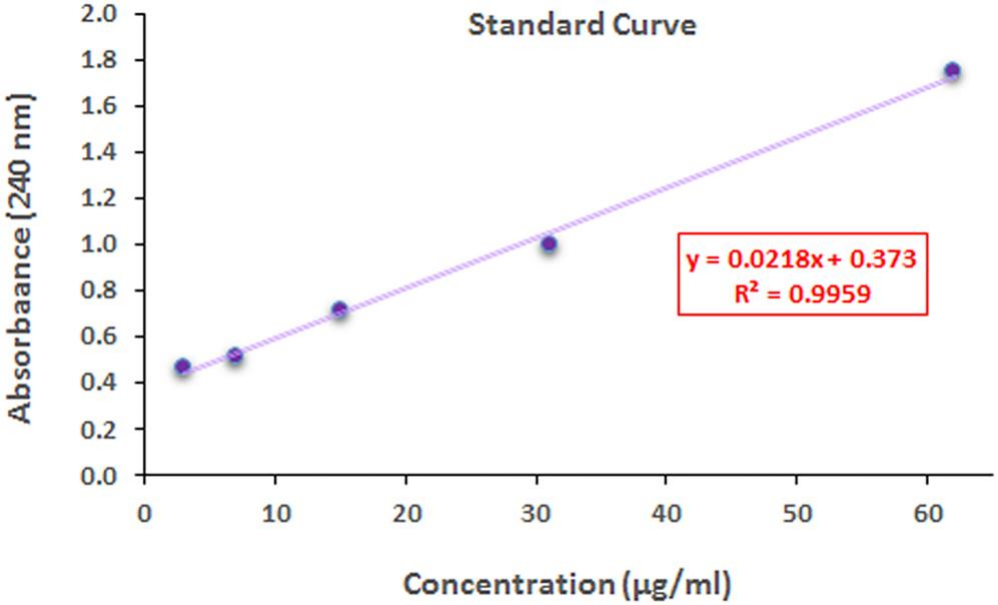
Standard curve concentration of dutasteride at the concentration ranging from 3 to 62 μg/ml.

The standard curve was drawn based on the serial dilution of dutasteride. As shown in Figure 1, the value of *R* ^2^ = 0.9955 was calculated, and the concentration equation was obtained in absorption wavelength (*y* = 0.0218x + 0.373). Samples that had adsorption above 2 were excluded from the study. The linear regression with the *R* ^2^ = 0.9955 indicates good linearity, ranging from 62 to 3 μg/ml (Figure 1).

### 3.2 Oil screening

**FIGURE 2:**
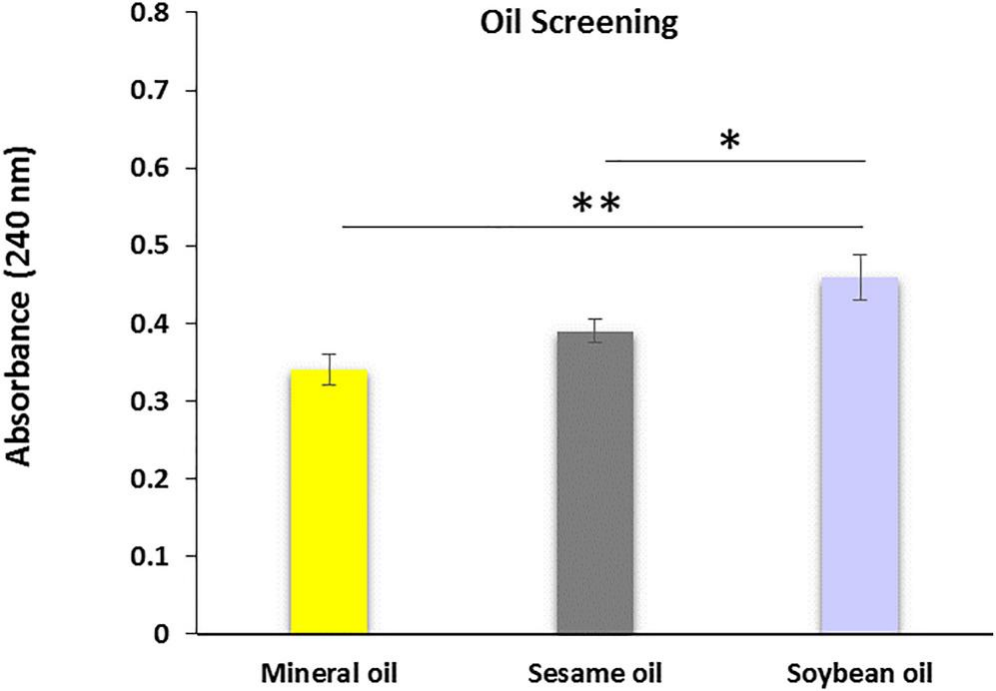
Oil screening for dutasteride nanocarrier preparation. * indicates (*p* < 0.05) and ** indicates (*p* < 0.01).

Dutasteride was dissolved in mineral oil, sesame oil, and soybean oil. The absorbance of the supernatant at 240 nm showed no significant difference between the solubility of dutasteride in mineral oil and sesame oil (*p* > 0.05). However, soybean oil significantly increased the dissolution of dutasteride than sesame oil (*p* < 0.05) and mineral oil (*p* < 0.01) (Figure 2).

### 3.3 Particle size and SPAN analysis

Five different dutasteride carriers were synthesised using soybean oil as the oil ingredient, tween 80 and span 80 as the surfactant, and ethanol as the co‐surfactant at different concentrations presented in Table 1. They were nominated A‐E. The Dynamic light scattering instrument and the SPAN formula evaluated hydrodynamic particle size and SPAN of dutasteride emulsions. Hydrodynamic particle sizes ranged from 5230 to 29.5 nm. Dutasteride emulsions of A and B were micrometre size; therefore, they were removed from the study. There was no significant difference between the hydrodynamic particle size of C and D dutasteride nanoemulsion (*p* > 0.05). Moreover, the dutasteride nanoemulsion of E had a significantly larger hydrodynamic particle size than B (*P* < 0.01) and C (*P* < 0.001). SPAN analysis showed a smaller SPAN for C and E dutasteride nanoemulsions than for D nanoemulsion (Table 1).

### 3.4 pH analysis

pH measurement data showed that there were no significant differences between the pH of C (5.29 ± 0.04) and D (5.25 ± 0.05) nanoemulsions (*p* > 0.05). Although the pH of nanoemulsion E (5.43 ± 0.07) was significantly higher than D nanoemulsion (*p* < 0.05), there were no significant differences between the pH of C and E nanoemulsions (*p* > 0.05).

### 3.5 Viscosity measurement

**FIGURE 3:**
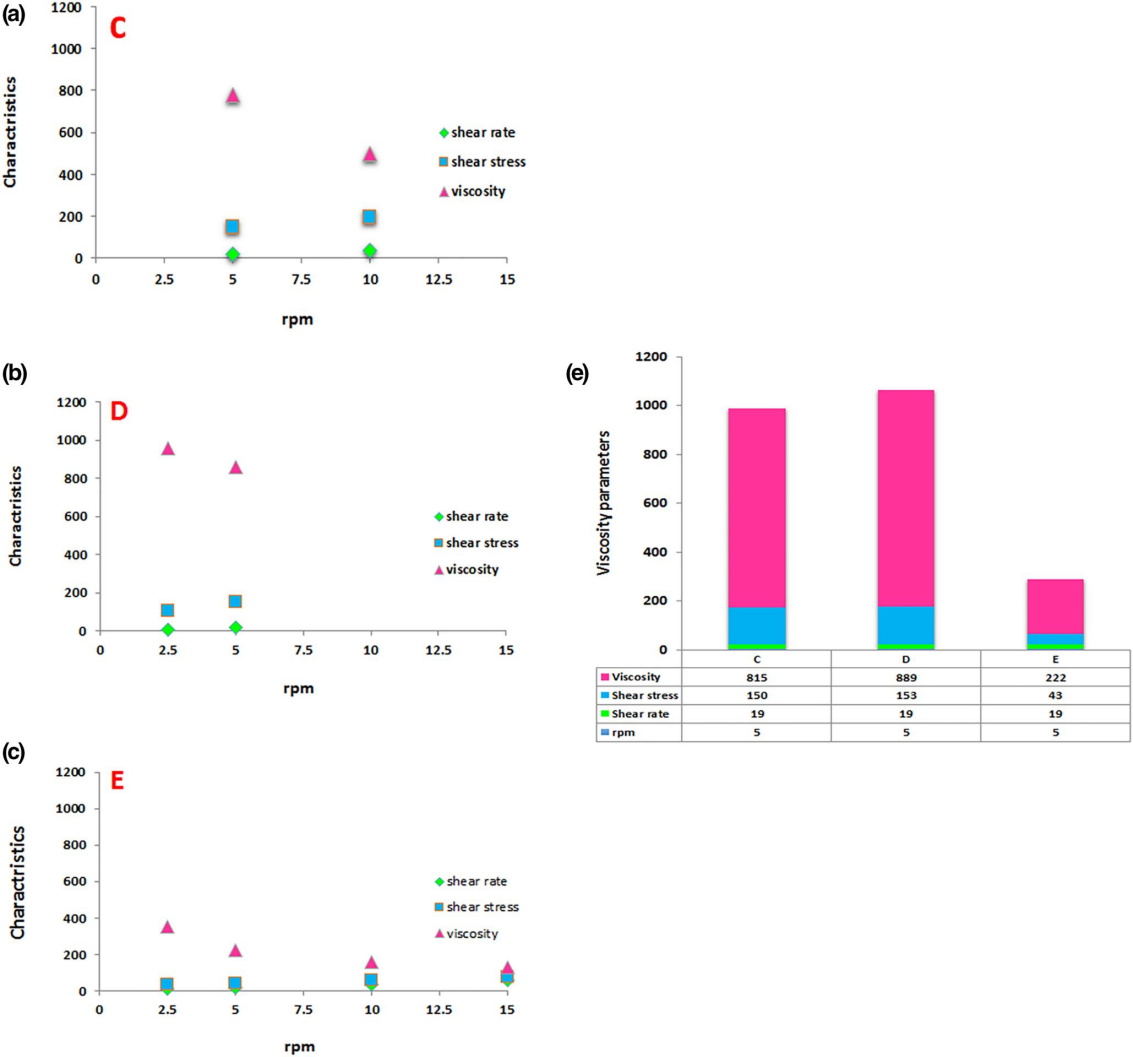
Viscosity evaluation of different dutasteride nanocarriers. a, b and c) Viscosity evaluation of C, D and E formulations. d) Viscosity evaluation of three formulations. Vertical axis units for shear stress, shear rate and viscosity are pascal (newtons per square metre), reciprocal seconds and newton‐second per square metre respectively.

The viscosity of nanoemulsions was measured at a shear rate of 9.5, 19, 38, and 57. C nanoemulsion showed a viscosity of 815 at a shear rate of 19 and 500 at 38, while the instrument with spindle 42 did not show measurable viscosity at a shear rate of 57. D nanoemulsion showed a viscosity of 889 at a shear rate of 19, while the instrument with spindle 42 did not show measurable viscosity at a shear rate of 38. E nanoemulsion showed a viscosity of 227 at a shear rate of 19, 158 at 38, and 131 at 57. Since all nanoemulsions showed measurable viscosity at a shear rate of 19, a single shear rate of 19 was selected as the reference to compare the viscosity of nanoemulsions in this study (Figure 3). Based on data from a shear rate of 19; there was no significant difference between the viscosity of C and D nanoemulsions (*p* > 0.05). At the same time, both of them showed significantly higher viscosity than E nanoemulsion (*p* < 0.001) (Figure 3).

### 3.6 EE% data

The EE% was measured using the ultrafiltration method and showed that EE% of C, D, and E nanoemulsions were 45.07% ± 0.31, 69.95% ± 0.48, and 99.9% ± 0.1. There was a significant difference between the EE% of all nanoemulsions (*p* < 0.001).

### 3.7 Thermodynamic stability evaluation

To select an ideal nanoemulsion, one of the most important characteristics is the thermodynamic stability of the nanoemulsion. In other words, thermodynamically unstable nanoemulsions were removed from the study. Following thermodynamic stability analysis of freeze‐thaw, centrifugation, and heating‐cooling, A dutasteride emulsion got biphasic at the centrifugation stage and was removed from the study. Although B, C, and D dutasteride emulsions were monophasic, their appearance was changed. Therefore, they were removed from the study. E dutasteride nanoemulsion was stable and went to further analysis (Table 2).

**TABLE 2:** Thermodynamic stability of dutasteride nanoemulsion

	Freeze‐thaw	Centrifugation	Heating‐cooling	Results
A	√	×	‐	Failed
B	√	×	‐	Failed
C	√	×	‐	Failed
D	√	×	‐	Failed
E	√	√	√	Passed

### 3.8 Ex‐vivo study: Permeation analysis

**FIGURE 4:**
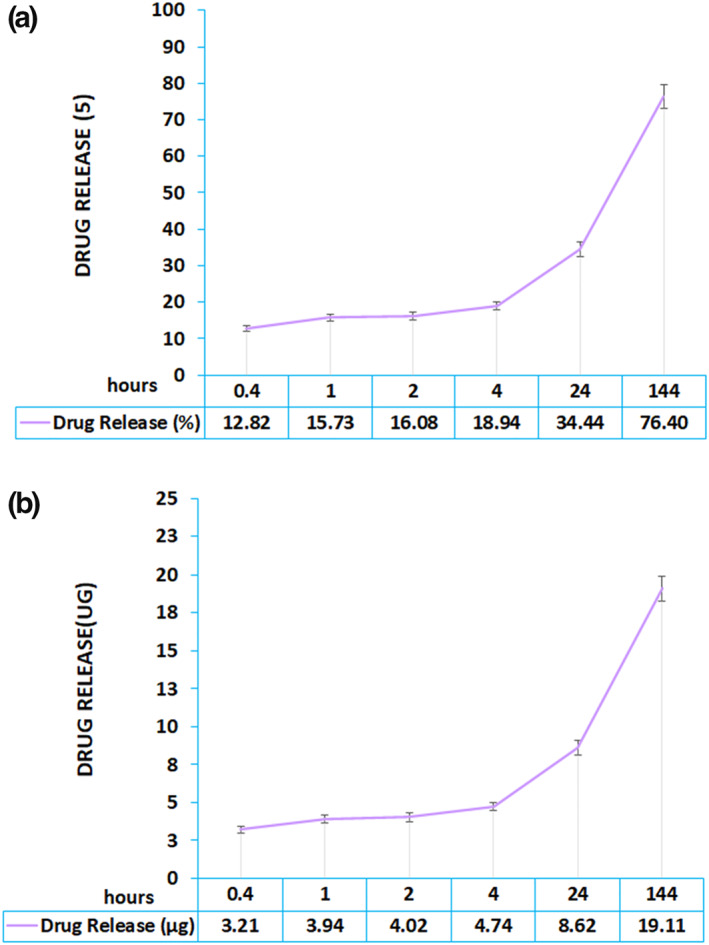
(a) Dutasteride release (%) profile of thermodynamic E nanocarrier through rat skin's abdomen. (b) Dutasteride release (μg) profile of thermodynamic E nanocarrier through rat skin's abdomen.

Based on thermodynamic stability findings, E nanoemulsion was selected for the ex vivo study of permeation analysis. Drug release and skin permeation analysis were evaluated using Franz diffusion cells. The dutasteride nanoemulsion's release profile showed a sustained dutasteride release at 24 h and continued for over 6 days. The figure shows that approximately 76% of dutasteride was released over 6 days (Figure 4a and b).

## 4 DISCUSSION

Hair loss or Alopecia is a treatable disorder that, over time, gets to chronically influencing a noteworthy part of the populace to decrease their self‐esteem. It is worth mentioning that the wealthy ancient Egyptians shaved their hair, and shaving was considered a symbol of wealth. Nevertheless, cultures have changed; now, thick hair symbolises beauty in many parts of the world. However, re‐styling hair and eyebrows may symbolise beauty in the following centuries. Nevertheless, in the present century, based on the criteria of beauty, let us look for a solution to this disorder. The present drug delivery system and formulations are not efficient enough to deliver the drug to the hair follicles and cannot release the drug in the hair follicles for a long time. Formulating and synthesising a drug delivery system based on nanocarriers would be beneficial and a gold strategy for a prolonged and sustained release drug delivery system in hair follicles. In the present study, a prolonged‐release nanocarrier of dutasteride was synthesised, characterised, and its influence on rat abdomen's skin was investigated. The concentration curve standard showed an *R* ^2^ = 0.9715, indicating a linear relationship between concentration and adsorption at 240 nm. Moreover, soybean oil is more solubilised dutasteride than other oils. Changes in synthesis parameters alter hydrodynamic particle size and other nanocarriers' characteristics, such as pH, thermodynamic stability, viscosity, and EE%.

As shown in the formulations of A and B, the lower ratio of S/Co made a larger particle size of dutasteride nanocarriers. When the ratio of surfactant to ethanol was increased from 1: 1–2: 1, particle size decreased from approximately 5230–4340 nm. Ethanol had enhanced hydrodynamic particle size; however, the increase of Smic also was critical in B nanocarriers. There are some controversial data related to ethanol and particle size. Tavakol et al. [28] and Ferreira et al. [29] demonstrated that the increase in ethanol had decreased the particle size of nanocarriers through decreasing interfacial tension. Some studies confirm our data related to an increase in particle size with an increase in ethanol concentration in nanocarriers [30, 31, 32, 33]. For example, Khodaee et al. showed that increased ethanol at constant temperature increased the particle size of mesoporous silica nanoparticles [30]. Another reason to increase particle size in our system with an increase in ethanol concentration would be that increased ethanol may, through non‐linear changes, not be thermodynamically ideal [34]. Besides, Chin et al. demonstrated that ethanol could increase surface fluidity resulting in intermicellar exchange kinetics [35]. Ostwald Ripening is another phenomenon involved in ethanol excess in the system. When the amount of ethanol is increased as high as the oil, it is solubilised, resulting in the formation of larger particle sizes from the small particles using the mass transport from dispersing phase to the continuous phase [36]. In summary, it might be said that optimum ethanol concentration is critical; particle size increases at higher ethanol concentration due to thermodynamic disturbance and Ostwald ripening, while particle size decreases due to decreasing interfacial surface. In our study, an increase of Smic to 40% and S/Co to 3: 1 were decisive in the particle size of dutasteride nanocarriers.

Another point relates to the increased surfactant and S/Co in the C nanocarrier. An increased surfactant through decreased surface tension results in smaller nanocarriers of approximately 32.4 nm. An increase in oil concentration from 5% to 10% did not significantly change hydrodynamic particle size and the pH of C and D nanocarriers. It seems that an increase of surfactant from 30% to 40% in A and C nanocarriers was sufficient to cover the surface of nanocarriers and decrease surface tension resulting in particle size of nanocarriers. The best formulations for hydrodynamic particle size were C, D, and E.

Data related to the viscosity of nanoemulsion showed that the increase of oil did not significantly change the viscosity of C and D nanocarriers and also did not significantly change the particle sizes. However, D nanocarriers with higher non‐significant viscosity than C nanocarriers had smaller particle sizes. Moreover, E dutasteride nanocarriers with significantly lower viscosity had larger hydrodynamic particle sizes. It is demonstrated that an increase in viscosity is inverse to the particle size of nanoemulsions [37, 38].

Based on the data derived from the EE% and particle size, and thermodynamic stability, it was conferred that larger nanocarriers of E nanocarriers had higher EE% than smaller nanocarriers. E nanoemulsion did not have ethanol and showed the highest EE%. It should be said that ethanol dissolves the drug and probably solubilise more drug in the nanocarrier; therefore, in the absence of ethanol, more drug can be kept in the nanocarrier, and its EE% has increased. Other nanocarriers of C and D showed less EE%, perhaps due to the rapid dissolution of dutasteride into ethanol during nanoemulsification; as a result, the accumulation of the drug inside the emulsion nanoparticles decreased and led to the drug leaving the internal phase, and the presence of the dutasteride in the dispersed phase.

The stability of nanocarriers was eventually owing to the electrostatic repulsion derived from deprotonated moieties. The optimum concentration of oil, surfactant, and co‐surfactant is decisive in guaranteeing the stability of nanoemulsion. Larger particles of A and B were thermodynamically unstable and could not pass the thermodynamic stability phase. Although based on Stokes' equation [39], the addition of ethanol probably through the reduction of interfacial tensions in nanoemulsion resulting in smaller particle size and stability of the system, in our study, E dutasteride nanoemulsion with larger particle size than C and D nanocarriers and smaller than A and B formulations, was more stable and passed all stage of thermodynamic stability. The stability of a system is based on free energy acquired through changes in interfacial free energy, inter‐droplet energy interaction, and entropy of dispersion [40]. Increased ethanol may, through nonlinear changes, not be thermodynamically ideal; therefore, E nanocarriers, with the optimised Smic and oil concentration, will be stable [34]. Based on these findings, E nanoemulsion was selected as the candidate nanocarriers for further analysis. Skin permeation analysis using Franz diffusion cells showed sustained release of dutasteride for 6 days. The first point is related to the permeation of dutasteride through the skin, showing the efficacy of dutasteride nanoemulsion in hair loss therapy; another critical point is that only 34% of dutasteride was released from the skin within 24 h. Nanoemulsion did not show a burst release during the first day, and 76% of dutasteride was released in 6 days. The sustained‐release profile of the developed dutasteride nanoemulsion was promising to deliver the drug to the hair follicles and release the drug in the hair follicles for a long time.

## 5 CONCLUSION

Dutasteride nanoemulsion was prepared using soybean oil, tween 80, and span 80, ethanol as a co‐surfactant, and different ratios of Smic and S/Co. Results showed that the lower ratio of S/Co made a larger particle size of dutasteride nanocarriers. Ethanol had enhanced hydrodynamic particle size. Particle size increases at higher ethanol concentration due to thermodynamic disturbance and Ostwald ripening, while particle size decreases due to the decreasing interfacial surface. An increased surfactant through decreased surface tension results in smaller nanocarriers of approximately 32.4 nm. In our study, an increase of Smic to 40% and S/Co to 3: 1 were decisive in the particle size of dutasteride nanocarriers. Dutasteride nanocarriers with significantly lower viscosity had larger hydrodynamic particle sizes. It is demonstrated that the increase in viscosity has an inverse correlation with the particle size of nanoemulsions. Other nanocarriers of C and D showed less EE%, perhaps due to the rapid dissolution of dutasteride into ethanol during nanoemulsification; as a result, the accumulation of the drug inside the emulsion nanoparticles decreased and led to the drug leaving the internal phase, and the presence of the dutasteride in the dispersed phase. The optimum concentration of oil, surfactant, and co‐surfactant is decisive in guaranteeing the stability of nanoemulsion. Although based on Stokes' equation, the addition of ethanol probably through the reduction of interfacial tensions in nanoemulsion results in smaller particle size and stability of the system. Skin permeation analysis using Franz diffusion cells showed sustained release of dutasteride in 6 days. The first point is related to the permeation of dutasteride through the skin, showing the efficacy of dutasteride nanoemulsion in hair loss therapy; another critical point is that only 34% of dutasteride was released from the skin within 24 h. Nanoemulsion did not show a burst release during the first day, and 76% of dutasteride was released in 6 days.

## AUTHOR CONTRIBUTIONS

Mehri Memar Bashi Aval and Saeedeh Saeedi synthesised the nanocarriers and characterised them, Elham Hoveizi wrote the manuscript, Reza Mombeiny and Mostafa Kazemi characterised the nanocarriers and performed Ex‐vivo study permeation analysis, Shima Tavakol got the grant, designed and supervised the research, and wrote the paper.

## CONFLICT OF INTEREST

The authors declare no conflict of interest.

## Data Availability

Data will be made available on reasonable request.
